# Predicting anti-PD-1 responders in malignant melanoma from the frequency of S100A9+ monocytes in the blood

**DOI:** 10.1136/jitc-2020-002171

**Published:** 2021-05-07

**Authors:** Soudabeh Rad Pour, Yago Pico de Coaña, Xavier Martinez Demorentin, Jeroen Melief, Manjula Thimma, Maria Wolodarski, David Gomez-Cabrero, Johan Hansson, Rolf Kiessling, Jesper Tegner

**Affiliations:** 1 Department of Medicine, Centre for Molecular Medicine, Karolinska Institutet, Stockholm, Stockholm, Sweden; 2 Department of Oncology-Pathology, Karolinska Institutet, Stockholm, Sweden; 3 Navarrabiomed, Complejo Hospitalario de Navarra (CHN), Universidad Pública de Navarra (UPNA), IdiSNA, Pamplona, Spain; 4 Biological and Environmental Sciences and Engineering Division (BESE), KingAbdullah University of Science and Technology KAUST, Thuwal, 23955, Saudi Arabia; 5 Karolinska University Hospital, Stockholm, Sweden; 6 Computer, Electrical, and Mathematical Sciences and Engineering Division (CEMSE), KingAbdullah University of Science and Technology KAUST, Thuwal, 23955, Saudi Arabia

**Keywords:** CD4-CD8 ratio, gene expression profiling, immunotherapy, active, therapies, investigational, programmed cell death 1 receptor

## Abstract

**Background:**

While programmed cell death receptor 1 (PD-1) blockade treatment has revolutionized treatment of patients with melanoma, clinical outcomes are highly variable, and only a fraction of patients show durable responses. Therefore, there is a clear need for predictive biomarkers to select patients who will benefit from the treatment.

**Method:**

To identify potential predictive markers for response to PD-1 checkpoint blockade immunotherapy, we conducted single-cell RNA sequencing analyses of peripheral blood mononuclear cells (PBMC) (n=8), as well as an in-depth immune monitoring study (n=20) by flow cytometry in patients with advanced melanoma undergoing treatment with nivolumab at Karolinska University Hospital. Blood samples were collected before the start of treatment and at the time of the second dose.

**Results:**

Unbiased single-cell RNA sequencing of PBMC in patients with melanoma uncovered that a higher frequency of monocytes and a lower ratio of CD4+ T cells to monocyte were inversely associated with overall survival. Similarly, S100A9 expression in the monocytic subset was correlated inversely with overall survival. These results were confirmed by a flow cytometry-based analysis in an independent patient cohort.

**Conclusion:**

Our results suggest that monocytic cell populations can critically determine the outcome of PD-1 blockade, particularly the subset expressing S100A9, which should be further explored as a possible predictive biomarker. Detailed knowledge of the biological role of S100A9+ monocytes is of high translational relevance.

## Introduction

Anti-programmed cell death receptor 1 (PD-1) immunotherapy aims to block the interaction of tumor-reactive T cells with PD-1 ligands (PD-L1 and PD-L2), which are expressed on numerous cell types, such as leukocytes and the tumor cells themselves, resulting in T-cell inactivation.^1 2^

Despite these encouraging results, the clinical outcomes of anti-PD-1 immunotherapy remain highly variable; only a fraction of patients display a durable clinical response and long-term progression-free survival (PFS), while the majority of patients either has no clinical benefit at all or experiences disease progression soon after an initial clinical response.^3–10^ Therefore, there is a clear need for predictive biomarkers and a more in-depth mechanistic analysis of the cellular populations involved in clinical response. Predictive biomarkers would enable selecting patients who are more likely to respond and provide predicted non-responders with alternative, perhaps more useful, therapeutic options. Single-cell analysis to assess PD-1 and downstream signaling molecules’ expression on tumor-infiltrating and circulating CD8+ T cells has proven beneficial to identify such predictive biomarkers.^11^ However, these approaches are impeded by the limited availability of patient material, a small number of parameters, the over-adaptation due to the absence of independent cohorts for validation, and the lack of systematic impartial bioinformatic pipelines.^12^

Here, we used peripheral blood mononuclear cells (PBMC) as an easily accessible source of immune cells. These samples were obtained from patients with metastatic melanoma (MM) before and during the treatment to examine immune signatures associated with responsiveness to anti-PD-1 immunotherapy.

We performed single-cell RNA sequencing of ~50,000 PBMC together with an in-depth immunomonitoring study and interactive bioinformatics pipeline to produce a thorough analysis of peripheral blood immune cells to identify response-associated predictive signatures.

## Materials and methods

### Patients

Collection of blood samples of patients with malignant melanoma undergoing treatment at Karolinska University Hospital occurred between January 2015 and September 2018. Patients received an infusion of nivolumab 3 mg/kg intravenously every 2 weeks, according to clinical routine, until disease progression or intolerable toxicity.

All patients had metastasized stage IV (defined according to the American Joint Committee on Cancer, AJCC) malignant melanoma (cutaneous or unknown primary), Eastern Cooperative Oncology Group (ECOG) performance status score of 0–2. Table 1 displays information about the patient cohort. Response evaluation was not strictly made according to immune-related Response Criteria (irRC); it was the responsible clinician who weighed clinical outcome and the radiological response.

**Table 1:** Patient characteristics

Patient characteristics	Discovery cohort (n=8)	Validation cohort (n=20)
	N (%)	N (%)
Age, median (range)	66 (45–83)	68.5 (37–82)
<65	4 (50)	8 (40)
>65	4 (50)	12 (60)
Gender		
Female	3 (37.5)	6 (30)
Male	5 (62.5)	14 (70)
Response		
CR	3 (37.5)	3 (15)
PR	1 (12.5)	5 (25)
SD	0 (0)	2 (10)
MR	0 (0)	2 (10)
PD	4 (50)	8 (40)
PFS		
>6 months	4 (50)	11 (55)
<6 months	4 (50)	9 (45)
BRAF status		
WT	5 (62.5)	10 (50)
V600	3 (37.5)	10 (50)
M stage		
M1a	3 (37.5)	7 (35)
M1b	3 (37.5)	5 (25)
M1c	2 (25)	8 (40)
Performance Status, ECOG (0–5)		
0	5 (62.5)	13 (65)
1	3 (37.5)	6 (30)
2	0 (0)	1 (5)

CR, complete response; ECOG, Eastern Cooperative Oncology Group; MR, mixed response; PD, progressive disease; PFS, progression-free survival; PR, partial response; SD, steady disease; WT, wild type.

The patients gave written informed consent to participate in blood sample collection connected with nivolumab treatment at the Department of Oncology, Karolinska University Hospital, Stockholm, Sweden.

### Assessments

Blood samples, including complete blood count, electrolytes, creatinine, liver, and a thyroid condition, along with the patients’ performance status and any adverse events, were assessed before each new dose was administered. Approximately 3 months after the last dose of nivolumab was administered, the patients underwent the first CT for tumor response evaluation. Radiological response evaluation was made according to irRC, although this was not part of a formalized research follow-up protocol.

### Isolation of peripheral blood samples

Peripheral blood samples were collected at three time-points during treatment and directly used for analysis. The first sample (baseline) was drawn immediately before the first nivolumab infusion; the second sample was obtained immediately before the third infusion (6 weeks). Pico de Coaña *et al*^13^ showed that some cell populations, such as myeloid derived suppressor cells (MDSCs), are sensitive to freezing. For this reason, the samples were stained and analyzed within 2 hours of blood extraction.

PBMC were isolated from blood samples by Ficoll density gradient centrifugation within 1 hour of sample collection, following the manufacturer’s instructions (Ficoll-Paque Plus, GE Healthcare Life Sciences, Sweden). As previously described,^13^ blood from two heparin tubes was pooled together, and Phosphate-buffered saline (PBS) (Gibco) was added to a final volume of 35 mL. Then, 10 mL of Ficoll-Paque was added to the tubes forming a layer of Ficoll below the blood. The samples were centrifuged for 22 min at 800g without acceleration and break. The PBMC layer was collected carefully and washed with PBS for 7 min at 450g to eliminate plasma and Ficoll leftovers. Subsequently, PBMC were washed twice with PBS for 7 min at 300g. Purified PBMC were counted and used immediately for flow cytometry staining and analysis or cryopreserved in fetal calf serum (FCS) (Gibco) with 10% dimethyl sulfoxide (Sigma Aldrich, Germany).

### Antibodies and flow cytometry

PBMC were stained immediately after purification as previously described.^14^ Dead cells were excluded using the LIVE/DEAD Fixable Aqua Dead Cell Stain Kit (Thermo Fisher Scientific). A detailed list of the antibodies used is provided in online supplemental table 2A. Stained PBMC was acquired using an LSRII (BD Biosciences) flow cytometer, followed by analysis in the FlowJo V.10.x platform (Treestar). In order to ensure consistent flow cytometer performance on a day-to-day basis, coefficient of variation (CV) values and signal-to-noise ratios were monitored using CS&T beads (BD Biosciences). A non-stained control was acquired for each sample, and critical stainings were validated using fluorescence-minus-one controls. Detailed gating strategies are shown in online supplemental table 2B and supplemental figure 9.

10.1136/jitc-2020-002171.supp1 Supplementary data

10.1136/jitc-2020-002171.supp2 Supplementary data

### Single-cell RNA sequencing of PBMC cell populations

Blood samples were collected from the eight patients with melanoma (before the first nivolumab infusion and before the third infusion (6 weeks)), PBMC were isolated, and cryopreserved until use. Before single-cell capturing, PBMC were thawed, washed once with RPMI 1640 medium and twice with PBS, suspended in PBS containing 0.04% BSA. We used 10× Genomics 3' v2 reagent kit (10× Genomics, Pleasanton, California, USA) for capturing single cells and library preparation. Single-cell barcoded cDNA libraries were quantified with Qubit dsDNA BR Assay Kit, diluted to 5 ng/µL, and re-measured with Qubit dsDNA HS Assay Kit (Invitrogen). We sequenced 98 bp read length and 50,000 reads/cell on HiSeq 2500 (Illumina, San Diego, California, USA).

The Cell Ranger Single Cell Software Suite was used to perform sample de-multiplexing, barcode processing, and single-cell 3′ gene counting.^15^ Reads were aligned to Ensembl human reference genome GRCh38, using STAR aligner. Graph-based cell clustering, dimensionality reduction, and data visualization were analyzed by the Seurat R package V.2.0 (.^16^ Cells that exhibited high transcript counts, >0.1% mitochondrial transcripts, were excluded from the analysis. During subset aggregation, libraries were normalized by log normalization using Seurat. Differentially expressed transcripts were determined in the Seurat R package utilizing the likelihood ratio test for single-cell gene expression statistical tests. Graphics were generated using the R packages ggplot2.

Gene Set Enrichment Analysis (GSEA) was performed on differentially expressed genes (DEGs). (http://www.broad.mit.edu/gsea/). A p value and a false discovery rate (FDR)^17^ were calculated to correct for multiple hypothesis testing. A gene set generally is considered significantly enriched when its *p* value is less than 0.05, and the FDR score is less than 0.25.^18^

### Statistical analyses

The analysis strategy was performed as previously described.^19^ Data sets resulting from flow cytometric analysis were checked for normality (Shapiro-Wilk test) and variance homogeneity (Bartlett’s test). A two-tailed t-test with Welch’s correction or non-parametric Mann-Whitney test (two-tailed) was used accordingly. Initially, median values within each population served as the cut-off points to determine high and low populations before survival analysis. In populations of interest, cut-off points were further refined using Cutoff Finder software.^20^ Overall, survival was calculated between the initial treatment and the time of death or last followed up. Kaplan-Meier analysis was used to calculate survival probabilities, and log-rank tests were used for curve comparison. The estimated cut-off points were also used for receiver operating characteristic curve analysis.

## Results

### Single-cell-derived stratification of responders versus non-responders to nivolumab using blood immune cells in patients with melanoma

**Figure 1:**
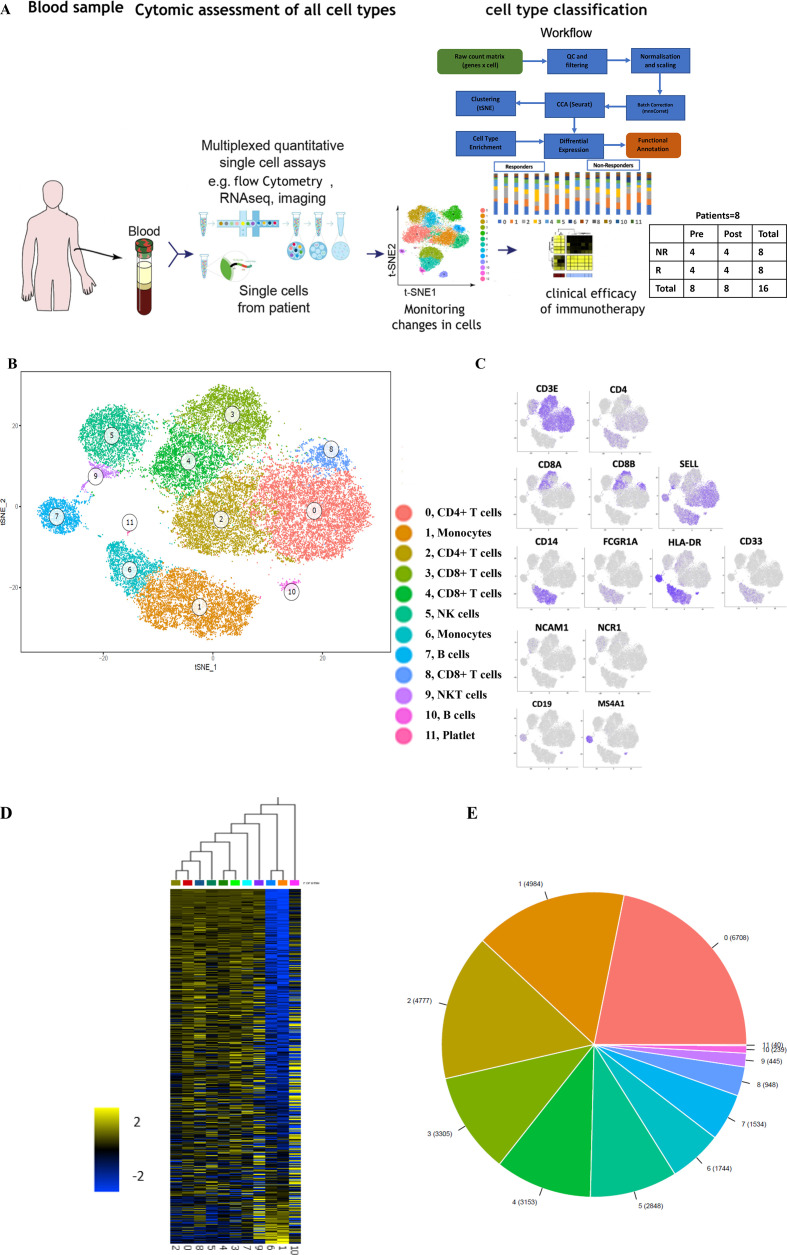
The immune landscape of PBMC from patients with melanoma treated with nivolumab (A) Study overview. (B) Eleven PBMC clusters. t-distributed stochastic neighbor embedding (t-SNE) of 48,000 PBMC, colored by clustering (C) or by expression (color BAR) of key cell type marker genes. (D) Cluster signature genes. Expression of top differentially expressed genes (rows) across the cells (columns) in each cluster. (E) The number of cells in each subset. Color bar as in (B). PBMC, peripheral blood mononuclear cells.

To analyze whether responsiveness to PD-1 checkpoint blockade is associated with specific characteristics of immune cells in the peripheral blood, we performed scRNA-seq on PBMC obtained at baseline or during immunotherapy from eight patients with MM undergoing first-line nivolumab treatment (figure 1A; table 1). The responses were classified as complete response (CR) and partial response (PR) for responders and progressive disease (PD) for non-responders.^21^ To relate molecular and cellular factors with therapeutic responsiveness, we classified each of the eight samples based on radiologic assessments into non-responders (n=4, including PD samples) or responders (n=4, including CR/PR samples) (table 1). Unsupervised clustering of 50,000 PBMC was performed on 50,000 PBMC to characterize their alterations on clinical intervention. This analyses identified 12 cell clusters with two B cell clusters (cluster 7 and 10), two monocytic clusters (1and 6), two CD4+ T-cell clusters (cluster 0 and 2), and five clusters enriched for CD8+ T/NK/NKT cells (3–5, 8–9) (figure 1B–E).

**Figure 2:**
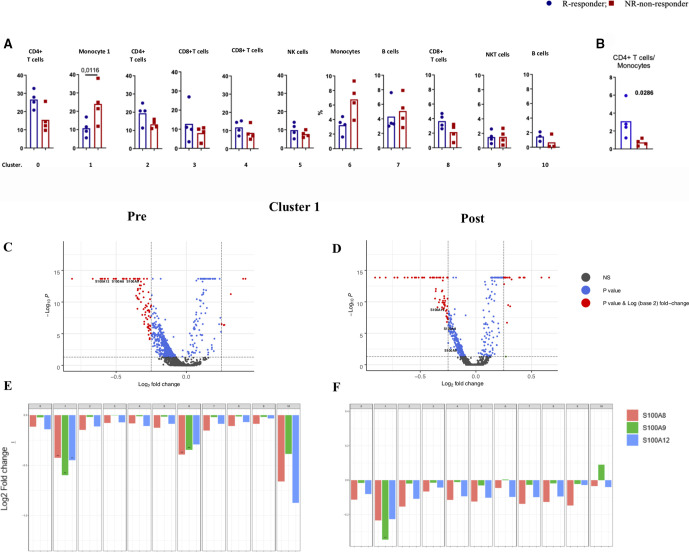
Characterization of monocytes and its association with clinical outcome. (A) Boxplot showing the frequency of each cluster in responders and non-responders, (B) CD4+ T cells/monocytes. (C, D) A volcano plot of DE genes based on a log-fold change in MM samples before (PRE) and during treatment (post). mRNAs that pass the cut-off p value <0.05 or p values corrected for FDA are represented in blue or red, respectively. S100A8, 9, and 12 are highlighted. (E, F) Bar plots of mRNA expression of S100A8, 9, and 12 in all clusters in pre (E) and post (F) samples. Cut-off p value <0.05 or p values corrected for FDA. DE, differentially expressed.

While each patient showed changes in cluster frequencies between baseline and post-treatment samples (online supplemental table 3, online supplemental figure 1A–C), there were no consistent changes when aggregating all samples. Interestingly, therapeutic non-responders had a higher abundance of cells in cluster 1 (p=0.0116; Tukey’s multiple comparisons test), which contains monocytic cells (figure 2A; online supplemental table 3, online supplemental figure 2). Following the distinct gene expression patterns in cluster 1 and cluster 6, they were designated monocyte 1 and monocyte 6, respectively (online supplemental table 4), based on immune cell type-specific gene markers (online supplemental table 5). Although the frequency of monocyte 6 was inversely associated with clinical response, this was not significant at the mRNA and protein level (figure 2A, online supplemental figure 3). Moreover, a higher ratio of CD4+ T cells (cluster 0) to monocyte 1 was associated with a better response (p=0.0286; Mann-Whitney test; figure 2B). Thus, our analysis enabled us to identify distinct characteristics of immune cell populations in peripheral blood of patients with melanoma associated with the clinical outcome of PD-1 checkpoint blockade.

### Differential gene expression in monocytes is associated with variable clinical responsiveness to nivolumab

Since we observed that responsiveness to PD-1 checkpoint blockade was associated with a lower frequency of monocytes in cluster 1, we set out to analyze these findings more in-depth. We further identified genes in monocyte 1 (online supplemental table 6) with significantly enhanced expression in non-responders, such as CH17-373J23, 1IFITM2, IFITM23, LY6E, CYBA, RPS4Y1, S100A8, S100A9, S100A12, HBB, CXCL8, IFI6, JUN, ANXA1, and KLF6. GSEA, which was performed on differentially expressed (DE) genes, identified a set of genes involves in the Toll-like receptors (TLR) receptor signaling cascade (FDR=1.77 e-4). The protein–protein interaction (PPI) network was generated using the STRING and Cytoscape by merging identified top DEGs (total node=20) to select the potential candidate for further evaluation. This analysis revealed that S100A9, CXCL8, and S100A8 have the highest biological interaction (online supplemental figure 4). Expression of S100 family members in tumors is an indication of more aggressiveness and metastasis,^22^ while their expression in myeloid cells is linked to hindered dendritic cell differentiation and augmented MDSCs formation^23 24^

We also found elevated expression of inhibitor of differentiation1 (ID1) in non-responders—post-treatment samples in cluster 1 (online supplemental figure 5A). Interestingly, monocytes in cluster 6 of non-responders were found to express higher levels of the transcription factor ETS2 (online supplemental figure 5B), a transcription factor known to promote survival of monocytes and macrophages.

In contrast, monocyte 1 in non-responders expressed less MT-ATP6, HLA-DQA2, TPT1, CD52, CXCR4, MAP3K8, IER3, HLA-DQB1, FOLR3, and TMEM176B (online supplemental table 6). Nuclear factor-κB (NF-kB) regulates these genes in response to TNF (FDR=1.36 e-2). It is known that NF-kB transcriptional factor activation in macrophages promotes suppressive phenotypes and increased cytotoxic T-cell infiltration to limit tumor progression.^25^

Collectively, these results indicated that S100A9 expression along with that of various other S100A proteins by monocytes in cluster 1 was one of the most differentially expressed genes between non-responders and responders to anti-PD1 therapy (figure 2C–F).

### Significance of higher CD4+/monocytes ratio in clinical responsiveness to nivolumab

Because we observed that responsiveness to PD-1 checkpoint blockade was associated with a higher ratio of CD4+ T cells (cluster 0)/monocytes (cluster 1) both at baseline and post-treatment, we set out to analyze these findings more in-depth. We found three responders had a CD4+ T cells/monocyte ratio over two and three non-responders a ratio below 1 (figure 2B). For this comparison, our calculations were conducted on cluster 0 and cluster 2 enriched for CD4 T cells separately with cluster 1 and cluster 6 enriched for monocytes. In contrast, CD8+ T cells/monocyte ratios did not differ between these two groups of patients. For this comparison, calculations were conducted on each cluster which 3, 4, and 8, were enriched for CD8+ T cells, while cluster 1 and cluster 6 were enriched for monocytes. On the contrary, CD4+ T cells in the responding patient group had higher expression of JUNB, SH3YL1, ZFP36L2, YPEL5, VAMP2, CXCR4, BTG2, KLF2, ZNF331, FOS, CCNI, TNFAIP3, FAM215B, BTG1, FTH1, NR4A2, GPR183l HNRNPH1, FOXP1, LTB, and DDX5 (online supplemental figure S4; online supplemental table 7). These genes are enriched for those regulated by NF-kB in response to TNF-alpha (FDR=3.22 e^−12^). Moreover, PPI analyses were generated using the STRING and Cytoscape by merging the identified top DEGs (total node=20), revealing that FOS, CXCR4, and FTH1 have the highest number of interactions when retrieved from the interrogation of the STRING database (online supplemental figure 6). Interestingly, it has recently been reported that combined treatment of novel CXCR4 antagonist with anti-PD-1 determined an increase of T cells/MDSC ratio and reduced tumor growth in the murine model.^26^

Furthermore, CD4+ T cells in the responding group had a higher expression of HLA-B, CD48, FOSB, CORO1A, IL27RA, ECI2, SHKBP1, PRPF38A, PGPEP1, and DDRGK1 (online supplemental table 8). This indicates enrichment in genes involved in the regulation of adaptive immune response (FDR=0.03). In contrast, CD4+ T cells in non-responders expressed more PPP2R2D, KPNA6, MYCBP, TRMT61B, RAB18, ARID3B, CSTB, EDEM3, IWS1, C8orf44, PIGL, and PDK3 (online supplemental table 9).

### An independent patient cohort confirms increased expression of S100A9 in monocytes of non-responders

Our results generated by scRNA-seq analysis of PBMC in an initial discovery cohort of patients with melanoma indicated that S100A9 expression by monocytes in cluster 1 was one of the most differentially expressed genes between responders and non-responders to anti-PD1 therapy. Most importantly, this revealed that low S100A9 levels in CD14+ cells were strongly associated with clinical responsiveness to anti-PD1 therapy, raising the question of whether S100A9 expression in monocytes could serve as a predictive biomarker for a clinical outcome of this type of treatment. In fact, this observation is very well in line with existing evidence that S100A9 may serve as a prognostic marker in cancer,^27^ as well as a novel marker for monocytic MDSC.^24 28^ Knowing this, we decided to perform flow cytometry analysis in an independent cohort of patients with MM (n=20) undergoing PD-1 blockade. We aimed to determine whether key observations made at the mRNA level by scRNA-seq analysis could be reproduced and validated at the protein level in a separate sample set. We studied frequencies of total monocytes and S100A9+ monocytes separately within the PBMC. The ratio of CD4+ T cell and monocyte frequencies and the ratio of CD4+ T cell to S100A9+ monocyte frequencies within the total PBMC were investigated.

**Figure 3:**
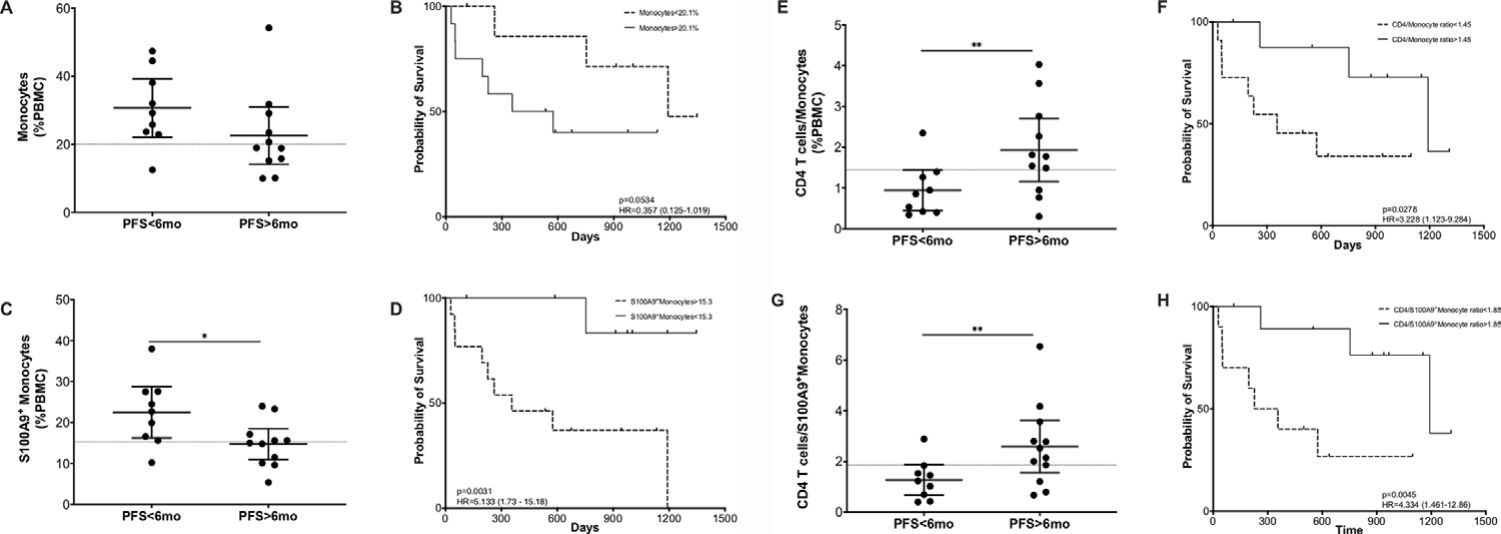
S100 A9 expression in monocytes and its association to clinical response to PD-1 blockade. Frequencies and Kaplan-Meier survival analysis of (A, B) monocytes (C, D) S100A9+ monocytes (E, F) CD4+ T cells/monocytes (G, H) CD4+ T cells/ S100A9+ monocytes in patients’ PBMC with long and short PFS at baseline. Each dot represent an individual patient, the dashed line represents the cutoff point that divides each parameter into high and low as calculated using Cutoff Finder software; mean±95% CI are represented. *p<0.05. PBMC, peripheral blood mononuclear cells; PFS, progression-freesurvival.

To enable objective group-wise comparisons for each of these parameters, we divided patients into two groups with PFS of either shorter (n=9) or longer than 6 months (n=11). In the patient group with longer PFS, three had CR, five PR, one MR, and two SD. Out of the nine patients with a shorter PFS, eight had PD and one MR. No significant differences in monocytes’ frequency were found between patient groups with long and short PFS (figure 3A). Also, with ±20.1% frequency difference in monocytes, no significant survival differences were observed figure 3B). Most importantly, when analyzing S100A9+ monocytes, we found that their frequencies were significantly higher in patients with short PFS (p value<0.001) (figure 3C). Moreover, patients with S100A9+ monocyte frequencies higher than 15.3% displayed a significantly lower overall survival when compared with those with frequencies lower than 15.3% (p value<0.05, HR: 3.228 (1.123–9.284)) (figure 3D). These differences were only significant before treatment, and no changes in these populations were observed as a result of PD-1 blockade therapy (online supplemental figure 7).

The relevance of monocytic populations was further confirmed when the CD4+ T cell/monocyte ratios were compared between long and short PFS patients (figure 3E). In this case, patients with prolonged PFS presented a significantly higher CD4+ T cell/monocyte ratio, resulting in an Overall survival (OS) advantage (figure 3F). These differences presented higher statistical significance when the CD4+ T cell/ S100A9+ monocyte ratio was considered (figure 3G, H). Overall, while the scRNA-seq data indicate that the frequency of monocytes is inversely correlated to overall survival, results from multicolor flow cytometric analysis in a more extended patient group suggest that it is the relative size of the S100A9+ monocyte subgroup within the total PBMC that is the strongest determinant of survival after anti-PD1 therapy.

To explore the possible relevance of different CD4+ T-cell memory subsets of Th1/Th2 subpopulations, an additional staining with CD45RA, CCR7, CXCR3, and CCR4 was carried out without finding significant differences (online supplemental figure 8).

A similar analysis was also done for ratios of all and S100A9+ monocytes with CD8+ T cells, but no differences were found.

## Discussion

Despite the considerable clinical success of the anti-PD1 therapy in MM, most patients do not experience clinical benefit. Therefore, we set out to identify predictive blood-based clinical biomarkers, which could pave the way for an increased understanding of underlying biology distinguishing between responders and non-responders. Using a single-cell genomics approach, we were able to identify the involvement of relevant cell types and thereby identify responders and non-responders. Thus, our analysis could detect a strong association of S100A9 expression in monocytes in clinical non-responders to anti-PD1 therapy, along with that of various other S100 proteins. Importantly, we could confirm these findings in an independent patient cohort, which indicated a strong association between the presence of high frequencies of S100A9+ monocytes and reduced PFS in patients with melanoma undergoing PD-1 checkpoint blockade.

In particular, our analysis revealed that both monocytes’ frequency or a lower ratio of CD4+ T cells to monocytes were inversely associated with overall survival. Similarly, S100A9 expression in the monocytic subset was inversely correlated with overall survival.

A substantial body of evidence highlights the importance of S100A9 in cancer. For example, a high density of S100A9-positive immune cells in tumor stroma of patients with prostate cancer ’ was correlated with poor clinical outcomes.^27^ Moreover, various studies in murine and human cells have provided strong evidence that S100A9 represents a novel marker for MDSC.^24 28^ In tumor cells, expression of S100A9 and that of other S100 family members lead to more aggressive outgrowth and metastasis.^22^ Most relevant for the current study is the finding in mice that expression of S100A9 in myeloid cells is associated with hampered differentiation of dendritic cells and enhanced MDSC formation.^24 29^ Moreover, the S100A8/9 heterodimer is overexpressed in MDSC in different types of cancer, and its expression is correlated with tumor load.^23 28–31^

We found elevated expression of ID1 and ETS2 in the monocytic population in non-responders’ post-treatment samples. This suggests a positive correlation between ID1, ETS2, and S100A8/9 expression. Interestingly, ID1 has been identified as an emerging phenotypic marker for monocytic MDSCs.^32^ Besides, the induction of ID1 favors tolerance and impaired immune responses by inhibiting myeloid cell maturation,^33^ which in turn suggests that ID1 may modulate MDSCs. For example, it has been shown that TGFβ-induced upregulation of ID1 led to MDSC expansion during tumor progression.^33^ This provides a potential explanation for the increased monocyte frequency in non-responders in our cohort.

Collectively, our results show elevated S100A9+ monocytes in PBMC of patients with MM non-responding to PD-1 inhibition and highlight the therapeutic potential of S100A9. Higher CD4+ T cell/monocyte ratios were further linked with better response to this therapy. Detailed knowledge of the functionality of S100A9+ monocytes is of high translational relevance. Thus, the monocytic population may be critical in the outcome of PD-1 blockade treatment, and expression of S100A9 proteins are possible predictive biomarkers.

## Data Availability

All data relevant to the study are included in the article or uploaded as supplementary information.
