# Supplementary material for: Predicting anti-PD-1 responders in malignant melanoma from the frequency of S100A9+ monocytes in the blood
Source: J Immunother Cancer. 2021 May 7;9(5):e002171. doi: 10.1136/jitc-2020-002171 (PMC8108662; doi:10.1136/jitc-2020-002171)
Supplement: Supplementary data [file jitc-2020-002171supp002.pdf]

BMJ Publishing Group Limited (BMJ) disclaims all liability and responsibility arising from any reliance placed on this supplemental material which has been supplied by the author(s)

Predicting anti-PD-1 responders in malignant melanoma from the frequency of S100A9+ Monocytes in Blood

BMJ Publishing Group Limited (BMJ) disclaims all liability and responsibility arising from any reliance placed on this supplemental material which has been supplied by the author(s)

Supplementary

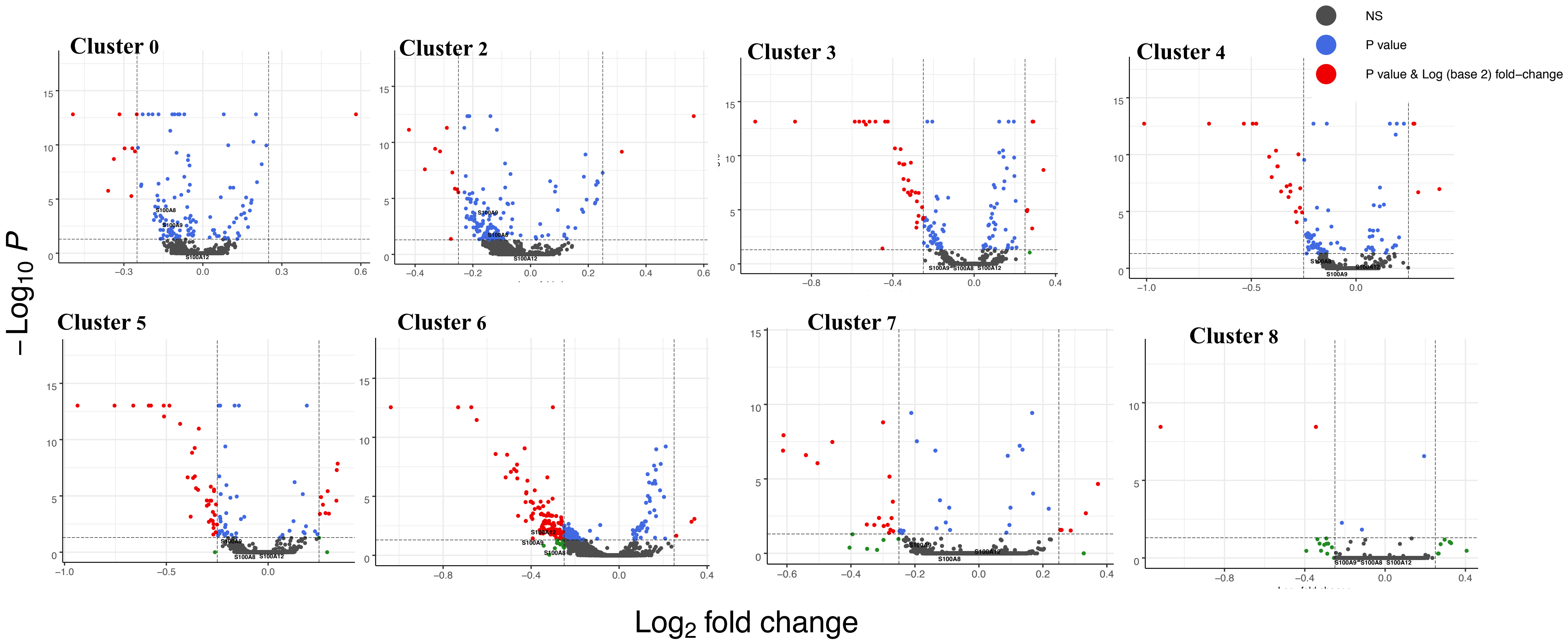

Supplementary Figure 1. Characterization of monocytes and its associated with clinical outcome

Volcano plots of DE analysis in two groups of responder and no-responders in pre-treatment , highlighted are differential expression genes using Cutoff *p*-value < 0.05 or *p*-values corrected for FDA.

Supplementary Figure 2. Frequency of each clusters in Pre-treatment CMM patients PBMCs

(A) Boxplot showing the frequency of each cluster in two groups responder and no-responder, (B) CD4+ T cells/Monocytes. (C) Volcano plot of DE genes based on log-fold change (FC) CMM samples during treatment (Post). mRNAs that pass the cutoff *p*-value < 0.05 or *p*-values corrected for FDA are represented in blue or red, respectively. S100A8, 9, and 12 are highlighted.

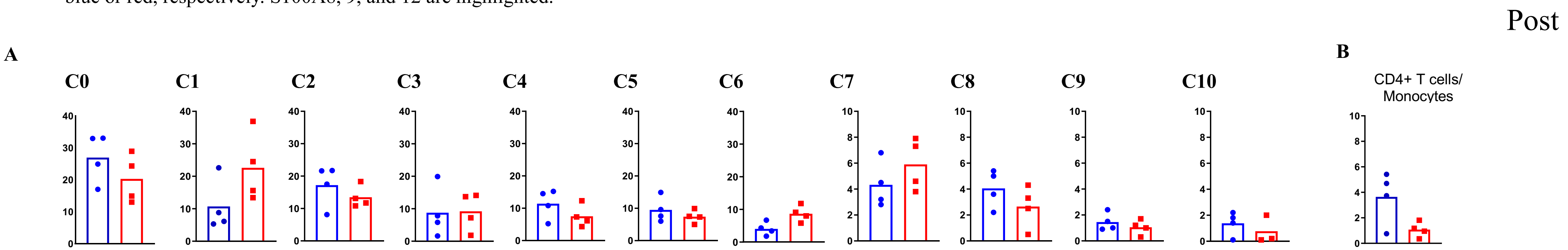

BMJ Publishing Group Limited (BMJ) disclaims all liability and responsibility arising from any reliance placed on this supplemental material which has been supplied by the author(s)

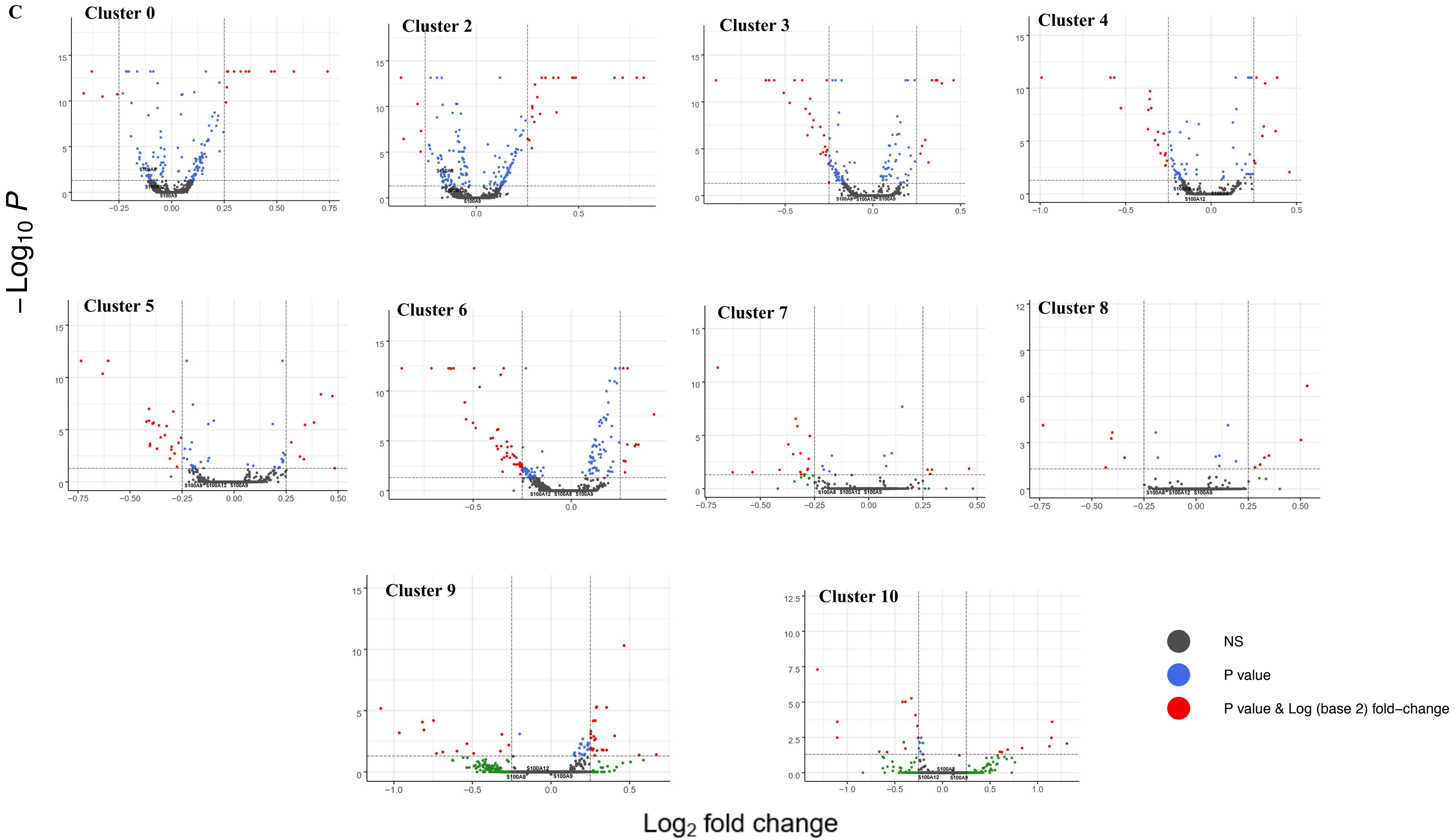

BMJ Publishing Group Limited (BMJ) disclaims all liability and responsibility arising from any reliance placed on this supplemental material which has been supplied by the author(s)

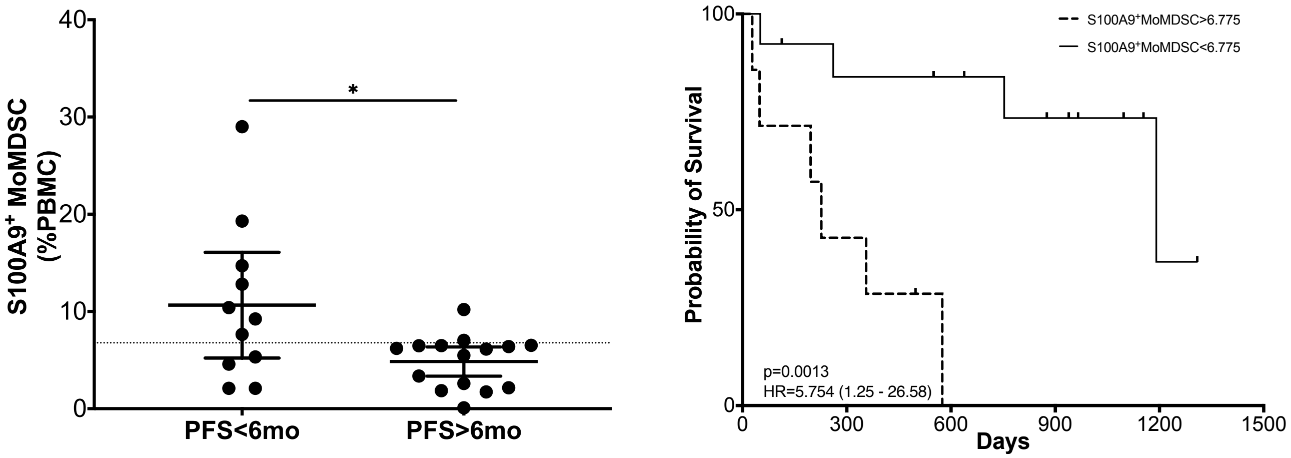

Supplementary Figure 3. S100 A9 expression in mo-MDSCs and its association to clinical response to PD-1 blockade

Frequencies and Kaplan-Meier survival analysis of mo-MDSCs in patients PBMCs with long and short PFS at baseline, each dot represents an individual patient, the dashed line represents the cutoff point that divides each parameter into high and low as calculated using Cutoff Finder software; mean ±95% CI are represented. *, $P<0.05$.

BMJ Publishing Group Limited (BMJ) disclaims all liability and responsibility arising from any reliance placed on this supplemental material which has been supplied by the author(s)

Supplementary Figure 4. Protein–protein interaction network between top DEGs in monocyters, obtained with Cytoscape v. 3.4.0 and retrieved from the interrogation of the STRING database. Nodes represents a molecular feature, and an edge represent the predicted functional associations between two markers (nodes). Degree measures biological interaction od each nodes and Betweenness centrality measures to identify essential nodes,

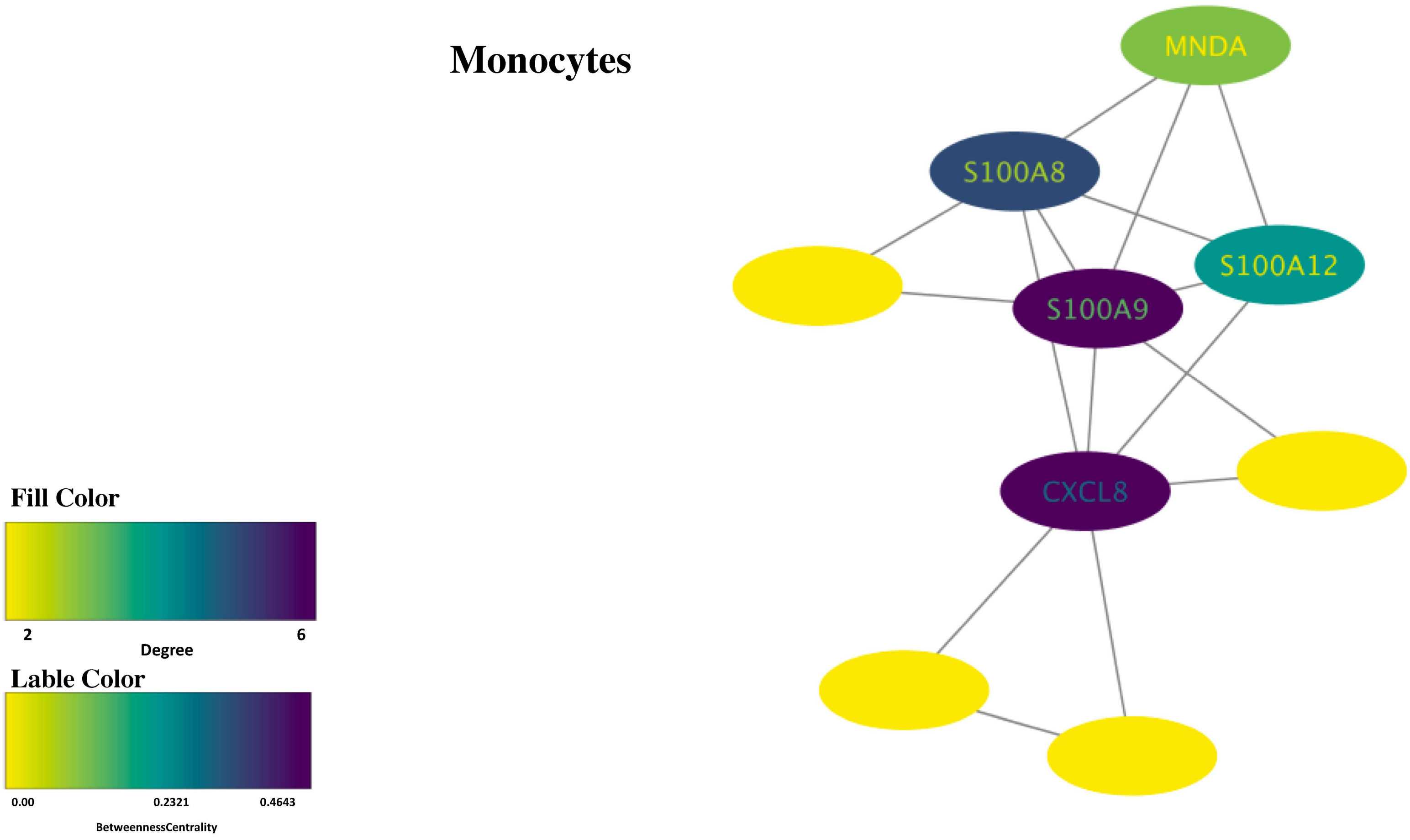

Supplementary Figure 5. ID1 expression in monocytes (A) bar plot of mRNA expression of S100A8,9,12, ID1, ETS2, THBS1 and RAP1A in all clusters in pre and **(B)** post samples. Cutoff *p*-value < 0.05 or *p*-values corrected for FDA.

A

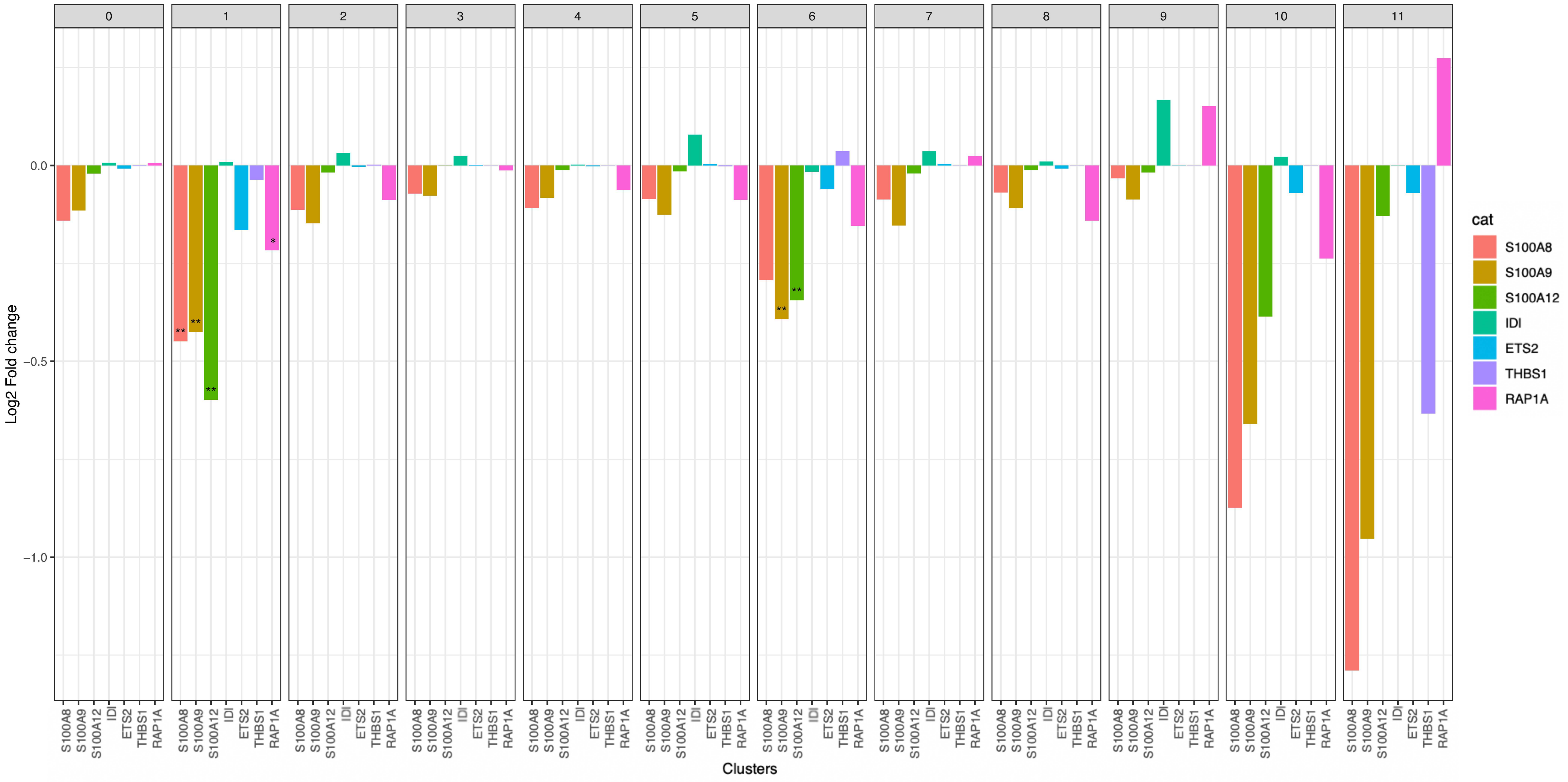

B

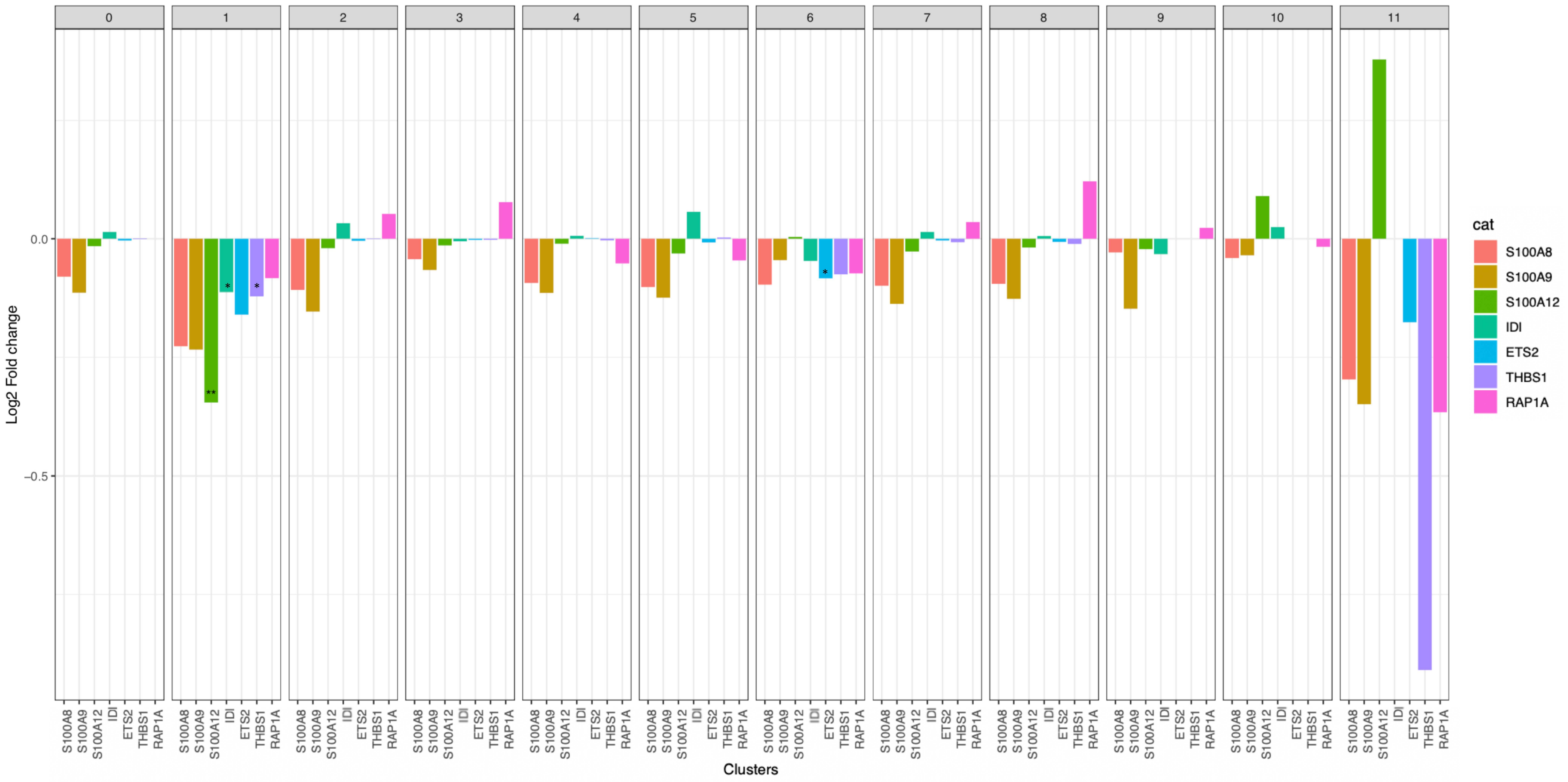
0
1
2
3
4
5
6
7
8
9
10
11
0.0
−0.5
Log2 Fold change
S100A8
S100A9
S100A12
IDI
ETS2
THBS1
RAP1A
Clusters
cat
**
*

Supplementary Figure 6. Protein–protein interaction network between top DEGs in CD4+T cells, obtained with Cytoscape v. 3.4.0 and retrieved from the interrogation of the STRING database. Nodes represents a molecular feature, and an edge represent the predicted functional associations between two markers (nodes). Degree measures biological interaction od each nodes and Betweenness centrality measures to identify essential nodes,

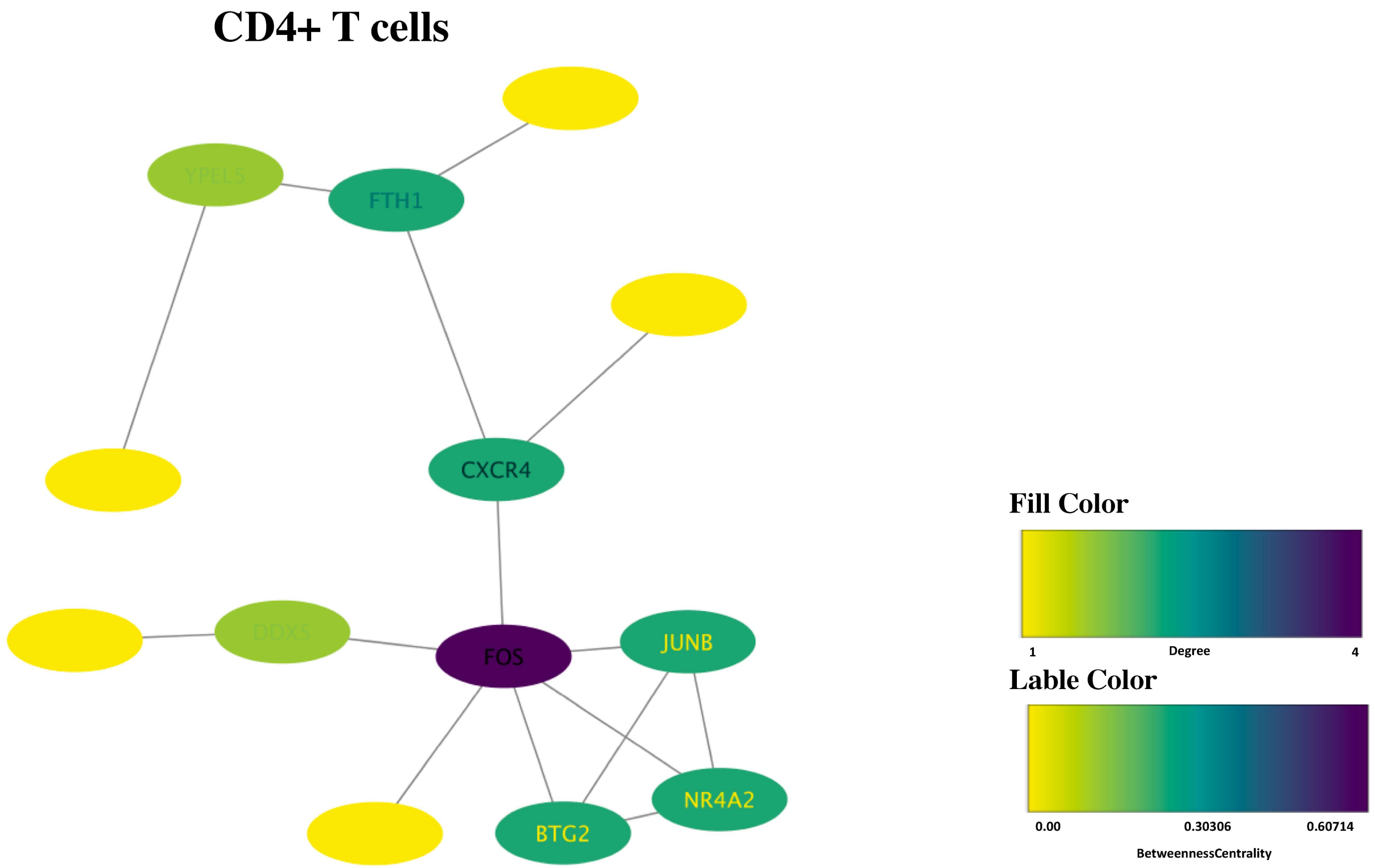

Supplementary Figure 7. Analysis of S100A9+ monocyte variation before and after nivolumab treatment

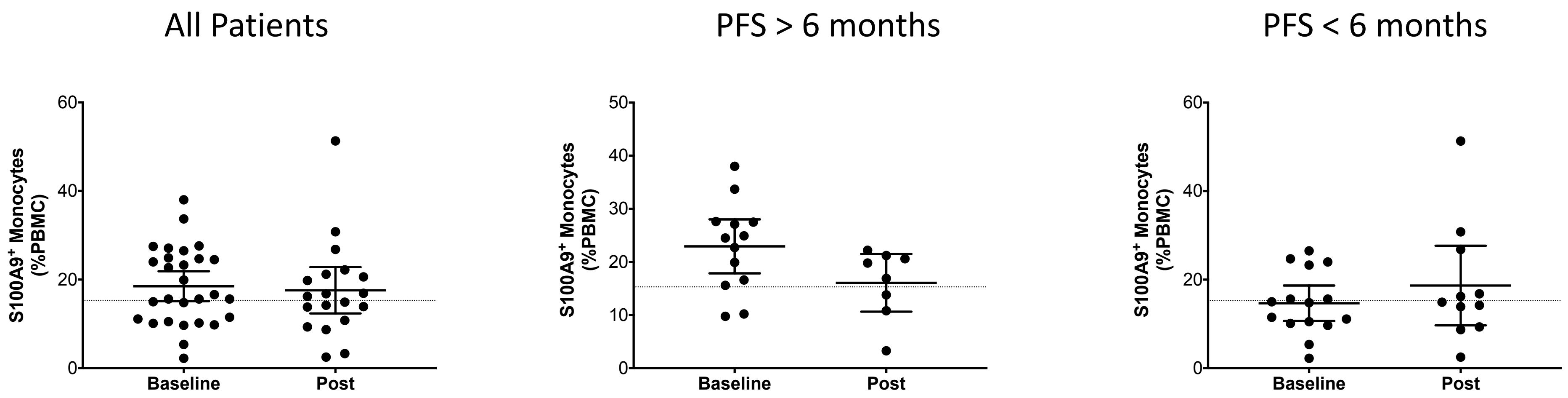

BMJ Publishing Group Limited (BMJ) disclaims all liability and responsibility arising from any reliance placed on this supplemental material which has been supplied by the author(s)

Supplementary Figure 8. Analysis of the CD4 memory subpopulations using CCR7 and CD45RA staining. According to this classification, naïve T cells are $CD45RA^{+}CCR7^{+}$, central memory (CM) T cells lose CD45RA expression but still home to the lymph nodes ($CD45RA^{-}CCR7^{+}$), effector memory (EM) T cells are $CD45RA^{-}CCR7^{-}$, and terminally differentiated T cells (EMRA) are $CD45RA^{+}CCR7^{-}$.

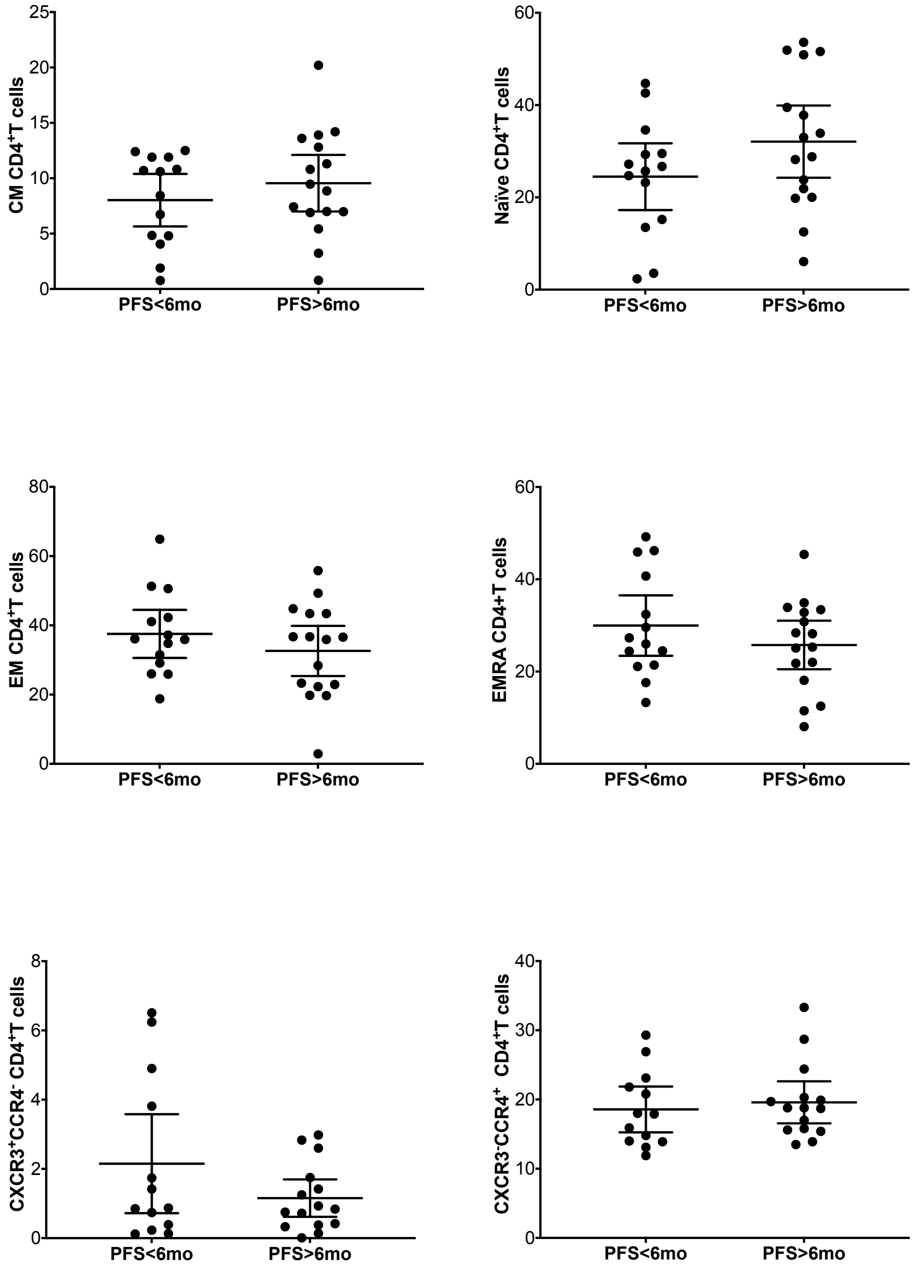

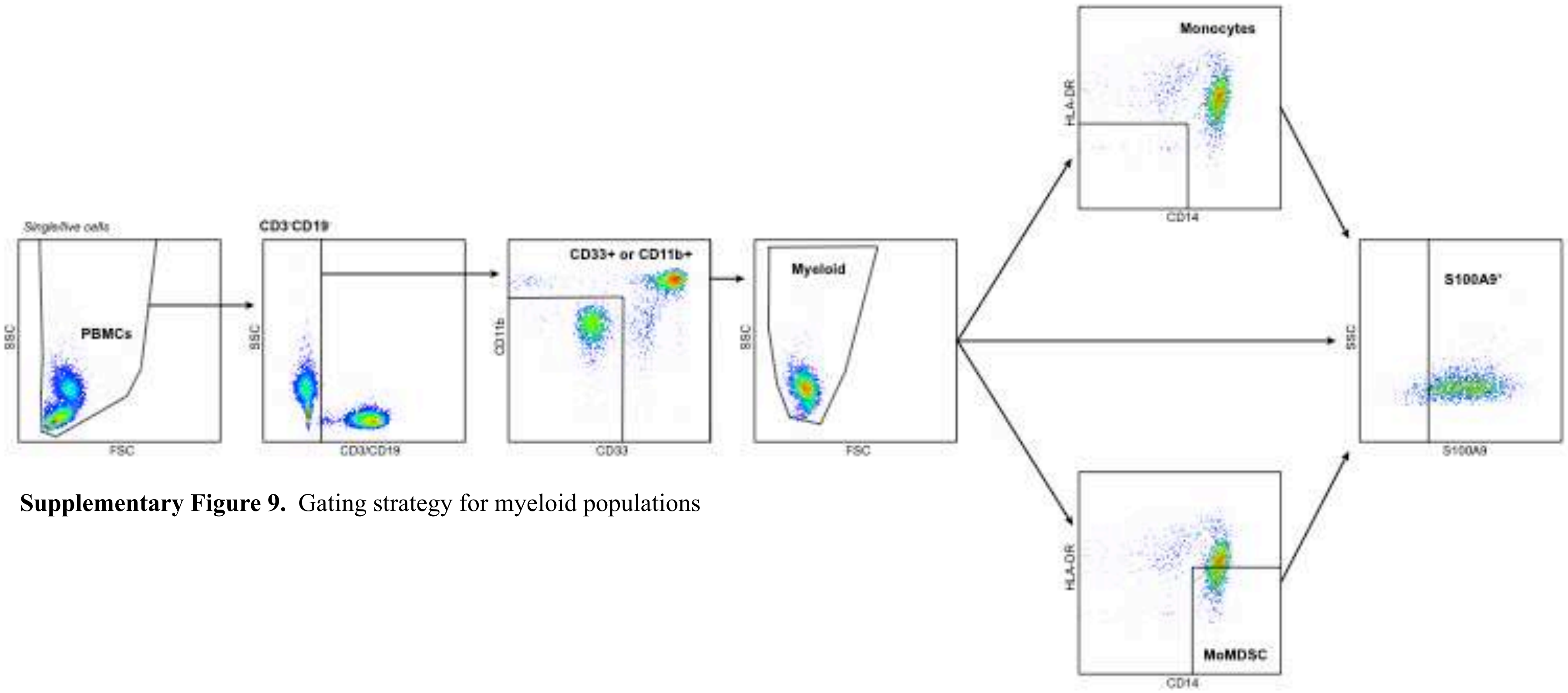

Supplementary Figure 9. Gating strategy for myeloid populations
